# Effect of Backward Walking on Attention: Possible Application on ADHD

**Published:** 2014-12-19

**Authors:** Davide Viggiano, Michele Travaglio, Giovanna Cacciola, Alfonso Di Costanzo

**Affiliations:** Dept. Medicine and Health Sciences, University of Molise, Campobasso (CB), Italy (davide.viggiano@unimol.it)

**Keywords:** *Froude number*, *gait analysis*, *motor control*, *ADHD*, *attention*

## Abstract

The human requires attentive effort as assessed in dual-task experiments. Consistently, an attentive task can modify the walking pattern and a attention deficit and hyperactivity disorder (ADHD) is accompanied by gait modifications. Here we investigated the relationships between backward walking and attentive performances in ADHD children (n=13) and healthy age-, height and weight matched controls (n=17). We evaluated the attentive/impulsive profile by means of a Go/No-Go task and the backward and forward gait parameters by step length, cadence and Froude number. Moreover, to test the causal relationship between attention and gait parameters, we trained children to walk backward. The training program consisted of 10 min backward walking session, thrice a week for two months. Results showed a significant negative correlation between Froude number during backward walking and reaction time in the Go/No-Go test. Besides, after training with backward walking control children increased their cadence by 9.3% and their Froude number by 17% during backward walking. Conversely, ADHD children did not modify their walking parameters after training, and showed a significant reduction in their number of errors in the Go/No-Go task (−49%) compared to the score before the training. These data suggest that specific physical training with attention-demanding tasks may improve attentive performance.

## I. INTRODUCTION

The human walking requires the integration of many sensorial feedbacks, which are used by multiple brain regions to modulate spinal circuits called central pattern generators [1]. This hierarchical control system produces a remarkable stability from one stride to the next notwithstanding the inverted-pendulum dynamic of the walking process [2]. Unusual walking patterns, however, may require additional attentive resources, as demonstrated by dual task experiments using a single-belt [3] or a split-belt treadmill [4] or standard walkers [5]. Similarly, the execution of the Stroop test, an attentive task, during walking induces a modification of the step width [6]. On the other hand, children with an impairment of attentive ability, such as in attention deficit hyperactivity disorder (ADHD), a frequent childhood behavioral disorder [7], may display modifications in the gait pattern, with increased in stride-to-stride variability [8]. Overall, a complex interplay emerges between neuronal networks regulating attention and those involved in gait modulation, suggesting the possibility to modify attentive performances through the execution of unusual gait patterns.

Backward walking (BW) is an unusual gait that does not involve a simple backward execution of the muscle activation patterns [9] and requires a separate neural control [10]. Therefore, it represents an interesting workbench to verify the possibility to influence attentive functions.

The possibility to improve attention through physical activity is very important because ADHD is treated mainly pharmacologically and new neuro-rehabilitation programs might increase the therapeutic options.

In this study we analyze the forward and backward gait of ADHD children and normal controls, before and after a backward walking training, and its relationship with attentive performances.

## II. METHODOLOGY

### Subjects and methods

#### Participants

Thirteen children (10 males and 3 females) who fulfilled the DSM-IV criteria for ADHD were included in the study. The presence of conduct, mood and/or anxiety disorders was an exclusion criterion. All children underwent a comprehensive neurological and mental examination. No children had a history of brain damage, epilepsy, psychosis or frank language disorder.

Control children were recruited from primary schools in the same geographical areas of ADHD children. The ADHD and controls were matched for age. Typically developing children were excluded from the study if: (i) the parent or the teacher stated that the child had ever received a clinical diagnosis (e.g. a behavioral/learning problem); or (ii) their full scale IQ estimate was below 80 as measured with the short version of the WISC-R. Table 1 provides the anthropometric data for all participants.

An additional group consisting of 32 control subjects, with the same characteristics described above, was also randomly assigne to two subgroups, one receiveing the backward training regimen (n= 16) and one who simply repeated the go/no-go task without any BW training just to evaluate the well known re-test effect (n=16).

The study has been approved by the local ethical committee and was carried out in accordance with the Declaration of Helsinki. Both children and parents gave informed consent to the work after the nature of the procedures had been fully explained.

### Go/No-Go task

The Go/No-Go task was conducted using a program running on a PC, separately for every subject. Subjects received at rest five runs each containing ten trials with 50% of go trials. During each trial, a rest period of random duration of 2–6 sec (during which children viewed a neutral target) was followed by a Go or No-Go signal (a round green target and a round checkerboard green-white target, respectively) pseudo-randomly interspersed.

Subjects were instructed to respond to the fully green target (Go signal) as fast as possible, by pressing a space bar button, and to withhold response when the green-white target (No-Go signal) appeared. They were presented with the green-white target 50% of the time, thus requiring response to half of the trials (Go trials) and response inhibition to the other half (No-Go trials).

The number of correct and incorrect (on No-Go signal) responses and their latencies were registered. The pressure of the bar before any Go/No Go signal was counted as measure of impulsiveness.

### Walking assessment

The gait was off-line quantified, after Go/noGo task, by calculating the step cadence, length and velocity while walking straight at a self-selected ‘natural’ speed over a delimited 10 meter pathway repeatedly in forward and in backward direction. A minimum of five trials in each walking direction was considered for quantification. Wireless accelerometers (based on the Wiimote remote controller, see [11]) were located on the leg to quantify the number of steps per unit time and the average velocity of each step; moreover, the gait was also recorded with a digital camera located on one side of the subject for off-line analysis. Step length was then normalized to subject’s height [7]. Furthermore, the Froude number was also calculated, a dimensionless index depending on walking speed and leg length according to the following formula:

Fr=v2/(l*g) [12]

Where v is the velocity, l is the leg length and g is the acceleration due to gravity. The Froude number has the advantage – compared to the walking speed - to be independent from age and body size.

### Training

To assess the possibility to improve attention with unusual gaits, all subjects were trained to walk backward for 10 minutes, thrice a week, for two months. Each subject self-selected the walking speed. At the end of the training period, subjects underwent again to the Go/No-Go task and to the gait assessment.

### Statistical analysis

Variables were examined for outliers and extreme values by box and normal quantile-quantile plots, and for distribution by Kolmogorov-Smirnov’s test. When normal distribution could not be accepted, variable transformations were reviewed. Data were analysed using repeated measures ANOVA to identify significant differences in gait parameters and attentive task before and after training. Linear regression analysis was carried out to identify correlations among gait parameters and results of the Go/No-Go task. The statistical significance level was set at α ≤ 0.05.

## III. RESULTS

**Figure 1.**
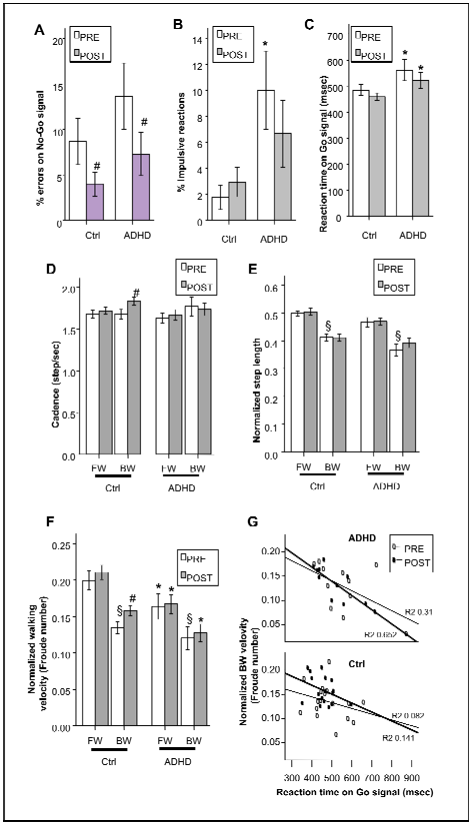
**Differences between ADHD children and normal control subjects for their performance on a Go/No-Go task (A–C), walking parameters (D–F) and response to a BW training**. Effect of a backward walking training on Go/No-Go task (A–C) and gait parameters (D–E) in children. During BW walking children reduce their speed (D) without significant changes in cadence (E); training with BW has significant effect on walking speed in BW (D) and on reaction time (C). F–G: correlation between reaction time and BW parameters before (PRE) and after (POST) training. *: p<0.05 ADHD vs control; # P<0.05 POST-training VS PRE-training; § p<0.05 BW vs FW.

Performance measures on the Go/No-Go task before and after BW training are presented in Fig. 1A–C. Before training, the percent of impulsive reactions was significantly greater (Fig. 1B; p=0.02) and the reaction time after Go signal was significantly slower (Fig. 1C; p=0.009) in ADHD children. The percent of errors was higher in ADHD group, but this difference did not reach the significance level. After BW training, both ADHD and control children reduced the number of impulsive reactions, errors (pressure on No-Go signal) and reaction time, but the the significance level was reached only in the ADHD group in which errors resulted smaller by 45.9% (p=0.019) (Fig. 1A–C).

The gait performance in both ADHD and control groups, before and after backward walking training are presented in Fig. 1D–F. Before the training, the cadence and the step length were not significantly different in the two groups. However, ADHD children showed a reduced Froude number in forward (FW) compared to normal controls by −17.6% (p=0.005 for group factor; two-way ANOVA; Fig 1F), that indicates the use of a sub-optimal velocity.

After backward (BW) training, subjects did not significantly modify their cadence (Fig 1D) neither their step length (Fig 1E). However, the cadence (+9.3%; p<0.05) and the Froude number (17%; p<0.05) were both significantly increased in control subjects while walking in backward direction. Conversely, ADHD children did not show any change in their gait parameters after BW training.

Further analysis of the correlation between the reaction time on a Go/No-Go task and the Froude number is represented in Fig 1F. Linear regression analysis showed significant negative correlation between the reaction time and the Froude number in ADHD children but not in control children. Moreover, after BW training the linear relationship between reaction time and Froude number was more significant and the beta coefficient in the linear model was more negative (beta=−0.56 before training and beta=−0.81 after training in ADHD children). The other parameters measured during the Go/No-Go task (e.g. % of errors and % of impulsive reactions) did not show significant correlations with gait parameters.

**Figure 2.**
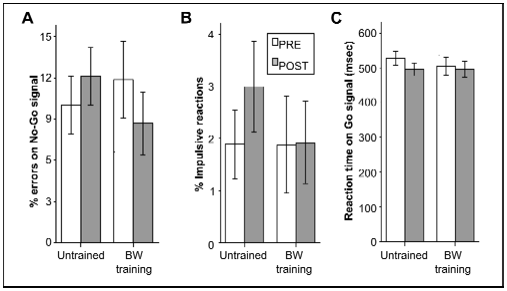
**Effect of BW training compared to no training on a Go/No-Go task in normal control subjects.** *Abstract* - The study evaluated the dietary habits in two groups of young athletes, practicing two different sports: soccer players and cycling. The dietary habits of 47 athletes were investigated by questionnaire. Body Mass Index, Fat Mass, Free Fat Mass, Total Body, Intracellular, Extracellular Water and Phase Angle were measured by bioimpedance. The *t*-Student test for unpaired data was used. Significance was set at P < 0.05. Body Mass Index was similar between the groups, while total body water and extracellular water were significantly higher in the soccer player group (soccer players: 63.8±1.96%; cyclists : 59.8 ± 8.7%; and soccer players 43.9±3.1%, cyclists 43.8 ±2.1%, respectively). Fatty mass of the soccer player group (14.5±2.9%) was significantly lower than that of the cyclist group (19.5±3.6%). Daily food intake was similar between the two groups (2844 kCal/die for soccer players /2630 kcal/die for cyclists), and lower than recommended. There was a low intake of Calcium (soccer players 1120±128.9 mg/die, cyclists 718±309 mg/die) for both groups, and a low intake of Potassium for soccer player (2576 mg/die ± 52.4) The caloric intake of adolescent athletes is lower than recommended. Body composition is significantly different between soccer players and cyclists.

To verify how much the modifications in the go/no-go task were imputable to the training regimen or to a re-test effect, an additional experiment was carried out with a group of subjects untrained and one trained with the backward walking protocol. Resutls are shown in Fig. 2 and the statistical analysis did not demonstrate any significant difference between the two groups. However, the new control group trained with BW protocol showed only borderline improvement (p=0.0915 one tailed paired samples t-test) of the % errors on No-Go signal (Fig 2A), with a trend similar to that reported in the first experiment (Fig. 1A).

## IV. DISCUSSION

The main findings of the present study are the reduction of errors in an attentive task after training with BW, and the correlation between reaction time in a Go/No-Go task and Froude number when walking in backward direction in ADHD children.

As summarized in Table 2, normal subjects, during a standard BW, use a smaller Froude number, that is a non-optimal velocity normalized by the leg length. ADHD children showed a further modification in their gait before training: a reduced Froude number when walking both FW and BW. In agreement with a previous report, ADHD children did not show other modifications in their gait [7]. The selection of a non-optimal Froude number during walking indicates an imbalance in the choice of the optimal velocity normalized by the leg length.

Interestingly, after training with BW, normal children improve their Froude number when walking BW, whereas ADHD children fail to show such a modification. Previous work already reported several changes in motor control occurring during backward walking [2,10]. Specifically, walking speed is reduced during backward walking, whereas cadence does not change [13]. This effect has received various interpretations. The possibility to modify the Froude number in BW to values closer to FW, after some training, suggests that the reduction of speed during BW is actually a reaction of the brain when performing this motor sequence, which is slowly learned through experience. Indeed, it is known that BW training may improve physiologic efficiency [14]. Our data show that BW training induces modifications in the reaction time in a Go/No-Go task, mainly in ADHD children. It is already known that locomotor activity improves reading comprehension and reaction times in a flanking task [15] and that simple walking improves concentration in children with ADHD [16].

Our observations on BW training are particularly interesting because this suggests a novel form of intervention for ADHD children, which might support neuro-rehabilitation and physical rehabilitation programs, see e.g. [17]. Moreover, the correlation between reaction times in attentive scores and Froude number in ADHD children suggests that training programs based on BW might be further optimized in terms of number, and duration of training sessions and their intensification during the training.

## V. CONCLUSION

There is a significant negative correlation between Froude number during backward walking and reaction time in the Go/No-Go test. After training with backward walking normal children increase their cadence by 9.3% and their Froude number by 17% during backward walking, whereas ADHD children failed to modify their walking parameters after training. After training with backward walking ADHD children showed a significant reduction in their number of errors in the Go/No-Go task (−49%). Overall these data suggest that specific physical training with attention-demanding tasks may improve attentive performance.

## Figures and Tables

**Table 1.** Summary of subject anthropometric data.

Group	n.	age ± SE	height (cm)± SE	Weight (kg)± SE
Control	6F+11M	12 ±0.2	152±2	48±2.6
ADHD	3F+10M	12±0.3	148±4.5	44±3.6

**Table 2.** Effects of main factors on attentive and gait parameters

**Factor**	**Significant effect**
Walking direction (backward walking)	Shorter step length Smaller Froude number
Experimental group (ADHD children)	Increased number of impulsive reactions Slower reaction times Smaller Froude number
BW Training	Increased cadence and Froudenumber only in normal children Lower number of errors in Go/No-Go task in ADHD and normalchildren (in normal children thisresult showed similar trend but wasnot significant in experiment 2)
